# Splenic FDG PET uptake and CT volume as prognostic biomarkers in diffuse large B cell lymphoma

**DOI:** 10.1007/s11547-026-02233-w

**Published:** 2026-06-20

**Authors:** Jacopo D’Argenzio, Nicolò Rampi, Jacopo Pozzi, Valentina Vespro, Matilde Pavan, Elena Casiraghi, Francesca G. Rossi, Angelo Castello, Laura. V. Forzenigo, Massimo Castellani, Massimo Zilocchi, Francesco Passamonti, Gianpaolo Carrafiello

**Affiliations:** 1 Postgraduate School in Radiodiagnostics, Università Degli Studi Di Milano, Via Festa del Perdono 7, 20122 Milan, Italy; 2 Hematology Department, Fondazione IRCCS Cà Granda Ospedale Maggiore Policlinico, Via Francesco Sforza 35, 20122 Milan, Italy; 3 Radiology Department, Fondazione IRCCS Cà Granda Ospedale Maggiore Policlinico, Via Francesco Sforza 35, 20122 Milan, Italy; 4 Università Degli Studi Di Milano, Via Festa del Perdono 7, 20122 Milan, Italy; 5 Nuclear Medicine Department, Fondazione IRCCS Cà Granda Ospedale Maggiore Policlinico, Via Francesco Sforza 35, 20122 Milan, Italy

**Keywords:** Spleen, Diffuse large B cell lymphoma, Positron emission tomography, Computed tomography, Prognostic Biomarkers

## Abstract

**Objectives:**

To evaluate whether baseline splenic ^18F-FDG PET uptake and CT-derived spleen volume predict early progression (≤ 36 months) and progression-free survival (PFS) in diffuse large B cell lymphoma (DLBCL), and whether they interact.

**Materials and methods:**

Retrospective cohort of adults with newly diagnosed DLBCL undergoing baseline ^18F-FDG PET/CT and CT. Splenic PET positivity was defined visually (uptake exceeding hepatic background); spleen volume was measured semi-automatically. Early progression/relapse was analysed as a 36-month milestone endpoint and PFS as time-to-event. Multivariable logistic and Cox models included IPI, sex, treatment, splenic PET, spleen volume, and a PET × volume interaction. A prespecified PFS sensitivity analysis re-included event-free patients censored before 36 months.

**Results:**

124 patients were included (68 men; median age 68 years [IQR 57–77]); 45/124 (36.3%) were PET-positive; median spleen volume was 3.13 dL (IQR 1.92–5.79). IPI predicted ≤ 36-month progression (OR 1.52; *p* = 0.021) and PFS (HR 1.44; *p* = 0.004). Splenic PET positivity predicted ≤ 36-month progression (OR 2.83; *p* = 0.048) but not PFS (*p* = 0.103). The PET × volume interaction was significant (OR 0.70; *p* = 0.021; HR 0.81; *p* = 0.033). In PET-negative patients, larger spleen volume predicted higher risk (OR 1.42; *p* = 0.027; HR 1.22; *p* = 0.042), whereas volume was neutral in PET-positive cases. In the PFS sensitivity analysis (*N* = 145), the interaction attenuated (HR 0.83; *p* = 0.057) and the PET-negative volume effect was borderline (HR 1.21; *p* = 0.053).

**Conclusion:**

Splenic PET positivity is a dominant risk marker in DLBCL in this cohort. CT-derived spleen volume may add conditional prognostic information in PET-negative patients, but the incremental value of the PET × volume interaction is modest and requires external validation.

**Supplementary Information:**

The online version contains supplementary material available at https://doi.org/10.1007/s11547-026-02233-w.

## Introduction

Diffuse large B cell lymphoma (DLBCL) is the most common subtype of non-Hodgkin lymphoma, representing approximately 30–40% of all cases [1]. The addition of rituximab to the standard CHOP chemotherapy regimen (cyclophosphamide, doxorubicin, vincristine, and prednisolone) has significantly improved outcomes [2]; nonetheless, 45–50% of patients experience relapse or progression despite first-line treatment [3].

The International Prognostic Index (IPI) remains a widely used risk stratification tool [4], yet the integration of imaging biomarkers may offer incremental prognostic value, particularly in uncovering occult disease burden.

[^18F]FDG PET/CT is the reference standard for initial staging and response assessment in DLBCL, per the Lugano classification [5]. Recent studies have shown that splenic involvement—defined by either topographic contiguity with disease sites or abnormal FDG uptake—predicts inferior progression-free and overall survival [6]. Moreover, diffuse splenic [^18F]FDG uptake has been linked to increased relapse risk even in patients achieving complete metabolic response [7], suggesting a possible pathophysiological role as a marker of systemic dissemination or inflammation [8].

However, the relevance of anatomical parameters such as CT-derived splenic volume remains largely unexplored, and it is unclear whether this metric can complement metabolic information or retain predictive value in PET-negative patients. This study aimed to evaluate the prognostic relevance of baseline splenic [^18F]FDG uptake and CT-derived volume in newly diagnosed DLBCL, and to assess the interaction between metabolic and anatomical imaging parameters in predicting relapse or progression.

## Materials and methods

### Study design and population

The study was performed in line with the principles of the Declaration of Helsinki and national regulations. Approval was granted by the Ethics Committee Lombardia 3 (Protocol ID: 6002_21.05.2025_P).; Individual informed consent was waived owing to its retrospective nature and anonymised data handling.

We carried out a retrospective, single-centre cohort study at a large tertiary care academic hospital. Consecutive adults with newly diagnosed diffuse large B cell lymphoma (DLBCL) staged between 18 July 2012 and 5 March 2024 were identified through the institutional lymphoma registry. The sample comprised all consecutive eligible patients diagnosed during the study window; therefore, no a priori power calculation was performed.

Inclusion criteria were: (1) histologically confirmed DLBCL; (2) baseline [^18F]-FDG PET/CT and contrast-enhanced whole-body CT within 1 month from diagnosis; and (3) available clinical and laboratory data sufficient to calculate the International Prognostic Index (IPI). For the fixed 36-month endpoint, event-free status required documented follow-up of at least 36 months; therefore, patients who remained event-free but were censored before 36 months were excluded from the primary analysis to avoid outcome misclassification. For sensitivity analyses of PFS, these patients were re-included and treated as right-censored at last contact.

Exclusion criteria were patients with known chronic liver disease, portal hypertension, or prior splenectomy; however, no patients met these conditions.

Of 175 screened patients, 51 were excluded (22 incomplete data, 29 inadequate follow-up), leaving 124 patients in the final cohort. For sensitivity analyses, we re-included 21 event-free patients with complete data who had been censored before 36 months, increasing the eligible cohort to 145 patients. This study adhered to the STROBE reporting guideline for observational studies.

### Endpoints

Two prespecified endpoints were analysed. First, early progression was modelled as a fixed-time binary outcome defined as progression or relapse within 36 months from baseline staging; patients without an event were required to have ≥ 36 months of follow-up to be classified as event-free. Second, progression-free survival (PFS) was defined as time from staging to first progression, relapse, death from any cause, or censoring at last follow-up. Progression events were defined according to Lugano 2014 criteria [5]. The 36-month cut-off was chosen because most DLBCL progressions/relapses occur within the first 2–3 years, after which the hazard declines [9]; this landmark is consistent with contemporary first-line DLBCL trials reporting early event endpoints (e.g. POLARIX, GOYA, REMARC) [10–12] and helps limit dilution from late competing deaths, particularly in older patients.

### Data collection and variable definitions

Clinical, laboratory, and imaging data were extracted from electronic medical records and the institutional PACS. The proportion of missing data was below 5% for all variables; therefore, a complete-case analysis was performed [13].

Baseline variables included sex, age, B symptoms, Ann Arbor stage, extranodal involvement, lactate dehydrogenase (LDH; dichotomised by institutional upper limit of 214 U/L) and IPI score (0–5) [4]. Histopathology was reviewed per the 2022 WHO classification [14] and grouped as DLBCL-not otherwise specified (NOS), high-grade B cell lymphoma (HGBCL), or other specific subtypes including T cell/histiocyte-rich (THCR-DLBCL), EBV-positive DLBCL, transformed follicular (tFL) or marginal-zone (tMZL), and primary cutaneous leg-type DLBCL. First-line therapy was categorised into three groups—standard, intensified, and reduced-intensity— based on regimen intensity; detailed treatment composition for each category is provided in Supplementary (Table S3).

### Imaging acquisition and spleen assessment

Baseline [^18F]FDG PET/CT examinations were assessed from the clinical staging report issued at baseline by board-certified nuclear medicine physicians (single-reader). PET/CT was interpreted at initial staging, before outcome ascertainment, with access to standard clinical referral information (diagnostic indication and staging context).

Splenic [^18F]FDG uptake—diffuse or focal—was considered positive when visually exceeding hepatic background [15], using a liver-referenced qualitative criterion consistent with established lymphoma PET interpretation frameworks [16]. Splenic volume was measured on contrast-enhanced CT using a vendor-provided semi-automated spleen segmentation workflow (syngo.via, Siemens Healthineers), generating an initial 3D spleen mask subsequently reviewed and manually refined by radiologists using standard editing tools. Two radiologists (> 5 years’ experience) independently segmented each spleen; if the volume ratio was outside 0.95–1.05, a third adjudicating radiologist (thoraco-abdominal radiology specialist, > 15 years’ experience) repeated the segmentation, and final contours were defined by consensus.

CT and PET/CT were acquired on institutional multi-detector CT scanners (SOMATOM X.cite [Siemens Healthineers] and SOMATOM Definition DS 128, [Siemens Healthineers]), and hybrid PET/CT systems (Biograph 64 TruePoint [Siemens Healthineers] and Biograph Vision 450 [Siemens Healthineers]). Iodinated contrast medium Iopamiro 370 (Bracco Imaging Spa) was used for contrast-enhanced CT.

### Statistical analysis

Statistical analyses were performed in *R* (v4.3.2). A two-sided *p* value < 0.05 was considered significant. Continuous variables were reported as medians with interquartile ranges (IQR); categorical as counts and percentages. Group comparisons used Pearson’s *χ*^2^ or Fisher–Freeman–Halton exact test (10,000 Monte Carlo replicates), and Mann–Whitney *U* tests for continuous or ordinal data. Univariate PFS comparisons employed log-rank tests. Univariate logistic regression (binary endpoint) and Cox proportional hazards regression (PFS) yielded odds ratios (OR) or hazard ratios (HR) with 95% CIs. To ensure comparability across endpoints, predictors associated with both the binary endpoint and PFS in univariable analyses (*p* < 0.05) were included. Individual IPI components (ECOG, stage, extranodal sites, LDH) were excluded to prevent over-parameterisation; treatment type was retained a priori as potential confounder.

Four prespecified multivariable models were fitted for each endpoint: M1 (full cohort); M2 (full cohort plus PET × volume interaction); M3 (PET-negative); M4 (PET-positive). To quantify the incremental value of the interaction term in Cox models, we compared M1 versus M2 using a likelihood-ratio test (1 degree of freedom) and reported AIC, BIC, and Harrell’s concordance index (C). To address potential bias from excluding event-free patients censored before 36 months, we performed a PFS sensitivity analysis re-including these patients as right-censored and refitting Cox models M1–M4 with identical covariates and specifications.

Logistic-model fit was evaluated with residual deviance, Akaike information criterion, McFadden *R*^2^, Hosmer–Lemeshow test, and variance-inflation factor. Discrimination metrics (AUC-ROC, AUC-PR, accuracy, sensitivity, specificity, PPV, NPV, F1-score) were bootstrapped with 1000 replicates. Cox models were assessed using Harrell’s concordance index and global likelihood-ratio, Wald and score tests; proportional hazards assumptions were checked with Schoenfeld residuals.

Inter-reader reproducibility of CT-derived spleen volume was quantified on the independent pre-adjudication measurements using a two-way random-effects intraclass correlation coefficient for absolute agreement, single measurement: ICC (2, 1) [17–19]. Uncertainty was expressed as 95% confidence intervals, estimated by paired bootstrap resampling at the subject level (20,000 resamples). Agreement was further characterised using the mean absolute percent difference (MAPD) and Bland–Altman analysis (mean bias and 95% limits of agreement) [20]. Reporting of reliability and agreement followed GRRAS recommendations [21].

### Threshold analysis

Clinically relevant spleen volume thresholds were explored using: (1) ROC analysis for the binary outcome (Youden’s index), and (2) the maximally selected rank statistic [22] (minimum group size ≥ 10%; 9999 Monte Carlo replicates) for PFS. These thresholds were exploratory and not included in inferential modelling to avoid data-driven bias.

## Results

### Baseline characteristics and outcome groups

**Fig. 1:**
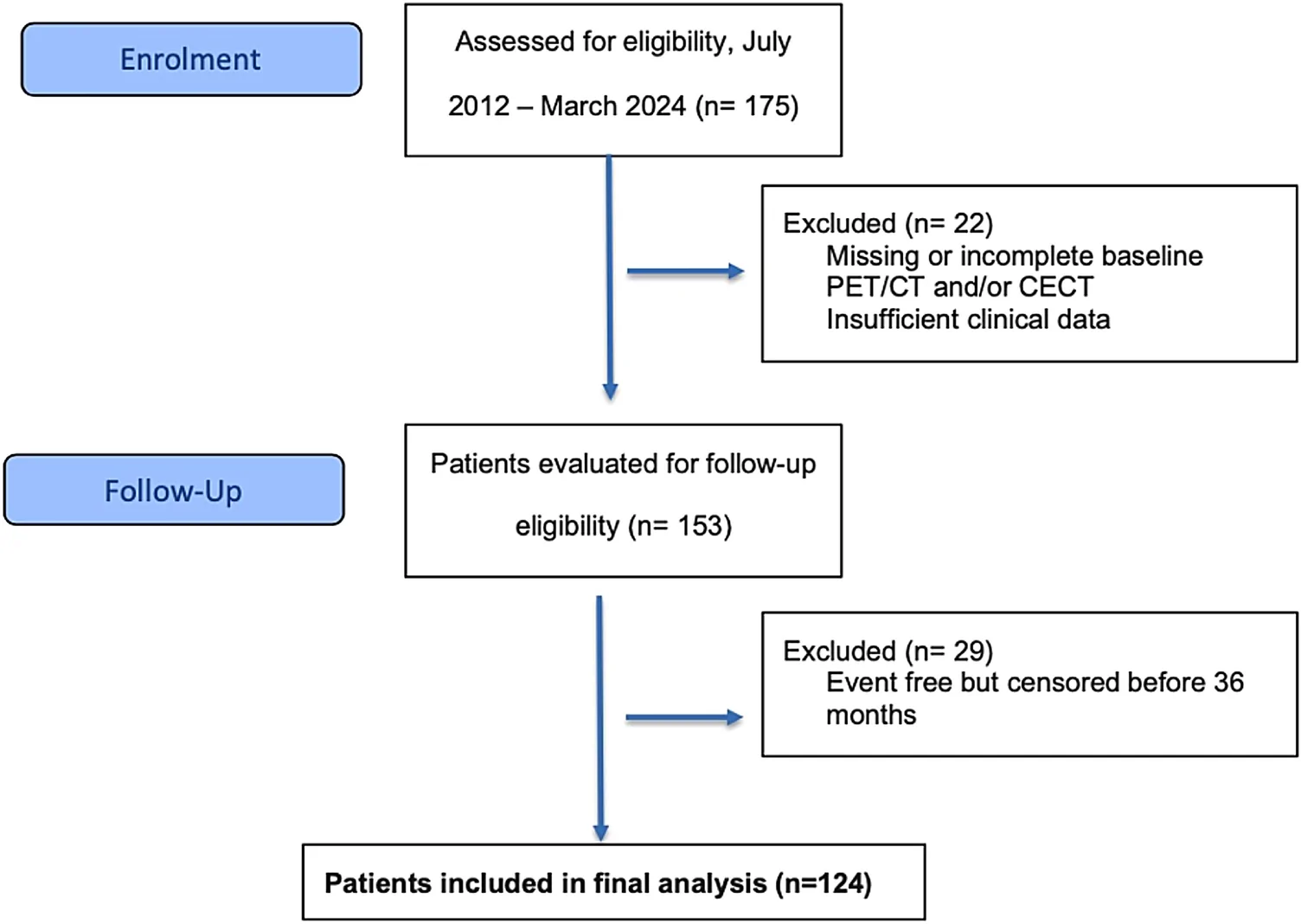
Patient inclusion flowchart. Flowchart showing patient screening, exclusion, and final cohort size. DLBCL = diffuse large B cell lymphoma; PET/CT = positron emission tomography/computed tomography; IPI = International Prognostic Index

After application of inclusion and exclusion criteria, 124 patients with newly diagnosed DLBCL were included in the final cohort (Fig. 1). Median age was 68.1 years (IQR 57.3–77.1); 68 patients (54.8%) were male. Most had advanced-stage disease (Ann Arbor III–IV, 87 [70.2%]), elevated LDH levels (85 [68.5%]), and B symptoms (45 [36.3%]). ECOG performance status was 0 in 63 patients (50.8%) and ≥ 2 in 24 (19.3%). Histologically, 103 (83.1%) had DLBCL-NOS, nine (7.3%) had high-grade B cell lymphoma (HGBCL), and 12 (9.7%) had other specific subtypes. Treatment intensity was standard in 105 (84.7%), intensified in 12 (9.7%), and reduced-intensity in seven patients (5.6%). PET-positive splenic uptake was detected in 45 (36.3%), and the median CT-derived splenic volume was 3.13 dL (IQR 1.92–5.79). Median follow-up was 66.7 months (IQR 48.2–77.2). Overall, 56 patients (45.2%) experienced progression or relapse, and 46 events (37.1% of the cohort) occurred within 36 months from baseline staging (Fig. S2). Baseline demographic and clinical characteristics, including comparisons by progression or relapse status, are presented in (Table 1 and Fig. S1). Events were significantly more common among males (66.1% vs. 45.6%, *p* = 0.04), patients with stage III–IV disease (87.5% vs. 55.9%, *p* < 0.001), elevated LDH (78.6% vs. 60.3%,* p* = 0.047), ECOG ≥ 2 (28.6% vs. 11.8%, *p* = 0.015), and IPI ≥ 3 (67.9% vs. 41.2%, *p* < 0.001). These variables were also associated with shorter PFS (all log-rank *p* < 0.034). Splenic involvement at baseline was more frequent among patients with disease progression, both in terms of metabolism (PET positivity: 51.8% vs. 23.5%, *p* = 0.002) and volume (median 4.21 dL [IQR 2.39–8.89] vs. 2.48 dL [IQR 1.67–3.86], *p* = 0.001). Both PET positivity and larger spleen volume were associated with inferior PFS (log-rank *p* < 0.047). PET-positive patients had substantially greater splenic volume compared to PET-negative ones (median 6.52 vs. 2.30 dL, *p* < 0.001) (Fig. S3).

**Table 1:** Baseline characteristics of the cohort with comparison by relapse status and univariate log-rank results for progression-free survival (PFS). Variables are reported for the full cohort (*n* = 124) and stratified by relapse status (Yes/No), with corresponding test statistics and two-sided p values

Variableᵃ	Full cohort (*n* = 124)	Progression/Relapse: YES (n = 56)	Progression/Relapse: NO (*n* = 68)	Between-groups tesᶜ	Statistic	p value	Log-rank *χ*^2d^	*p* value
Sex				Chi-squared	*X*^2^ = 4.41	0.036	6.12	0.013
Female	56 (45.2%)	19 (33.9%)	37 (54.4%)					
Male	68 (54.8%)	37 (66.1%)	31 (45.6%)					
B symptoms				Chi-squared	*X*^2^ = 2.46	0.117	4.43	0.035
No	79 (63.7%)	31 (55.4%)	48 (70.6%)					
Yes	45 (36.3%)	25 (44.6%)	20 (29.4%)					
Splenic PET positivity				Chi-squared	*X*^2^ = 9.42	0.002	11.49	< 0.001
No	79 (63.7%)	27 (48.2%)	52 (76.5%)					
Yes	45 (36.3%)	29 (51.8%)	16 (23.5%)					
Treatment intensity				Fisher (Monte Carlo)	–	0.081	20.15	< 0.001
Standard	105 (84.7%)	44 (78.6%)	61 (89.7%)					
Intensive	12 (9.7%)	6 (10.7%)	6 (8.8%)					
Reduced	7 (5.6%)	6 (10.7%)	1 (1.5%)					
Histological subtype				Fisher (Monte Carlo)	–	0.079	9.04	0.011
DLBCL-NOS	103 (82.1%)	44 (78.6%)	59 (86.8%)					
HGBCL	9 (7.3%)	3 (5.4%)	6 (8.8%)					
Others	12 (9.7%)	9 (16.1%)	3 (4.4%)					
Extranodal				Chi-squared	*X*^2^ = 0.03	0.863	0.00	1.000
No	43 (34.7%)	17 (30.4%)	26 (38.2%)					
Yes	81 (65.3%)	39 (69.6%)	42 (61.8%)					
LDH level				Chi-squared	*X*^2^ = 3.95	0.047	4.50	0.034
Normal	39 (31.5%)	12 (21.4%)	27 (39.7%)					
Elevated	85 (68.5%)	44 (78.6%)	41 (60.3%)					
IPI score				Mann–Whitney	*U* = 1256.5	0.001	14.70	0.010
0	7 (5.6%)	1 (1.8%)	6 (8.8%)					
1	17 (13.7%)	2 (3.6%)	15 (22.1%)					
2	34 (27.4%)	15 (26.8%)	19 (27.9%)					
3	36 (29.0%)	20 (35.7%)	16 (23.5%)					
4	22 (17.7%)	14 (25.0%)	8 (11.8%)					
5	8 (6.5%)	4 (7.1%)	4 (5.9%)					
Baseline spleen volume (dL)ᵇ	3.13 (IQR 3.87)	4.21 (IQR 6.50)	2.48 (IQR 2.19)	Mann–Whitney	*U* = 1218.0	0.001	3.94	0.047
Age at staging (years)ᵇ	68.05 (IQR 19.8)	68.2 (IQR 21.4)	68.05 (IQR 18.3)	Mann–Whitney	*U* = 1843.5	0.763	0.05	0.828
Ann Arbor stage				Mann–Whitney	*U* = 1316.5	0.001	12.88	0.005
I	17 (13.7%)	3 (5.4%)	14 (20.6%)					
II	20 (16.1%)	4 (7.1%)	16 (23.5%)					
III	17 (13.7%)	10 (17.9%)	7 (10.3%)					
IV	70 (56.5%)	39 (69.6%)	31 (45.6%)					
ECOG status				Mann–Whitney	*U* = 1458.0	0.015	25.10	< 0.001
0	63 (50.8%)	23 (41.1%)	40 (58.8%)					
1	37 (29.8%)	17 (30.4%)	20 (29.4%)					
2	20 (16.1%)	12 (21.4%)	8 (11.8%)					
3	4 (3.2%)	4 (7.1%)	0 (0.0%)					

ᵃValues are n (%) unless otherwise specified

ᵇContinuous variables are reported as median (interquartile range, IQR)

ᶜBetween-group comparisons: chi-squared or Fisher’s exact test for categorical variables; Mann–Whitney U for continuous/ordinal variables

ᵈUnivariate associations with PFS were evaluated by the log-rank test; *χ*^2^ statistics and p values are reported for each variable. For log-rank analyses, age and baseline spleen volume were dichotomised at the cohort median

DLBCL-NOS = diffuse large B cell lymphoma, not otherwise specified; HGBCL = high-grade B cell lymphoma; ECOG = Eastern Cooperative Oncology Group; IPI = International Prognostic Index; LDH = lactate dehydrogenase; IQR = interquartile range

### Inter-reader reproducibility of splenic volumetry

On pre-adjudication independent spleen volume measurements (*N* = 40 subjects; *k* = 2 readers), inter-reader reliability was excellent (ICC = 0.912, 95% CI 0.857–0.952). The mean absolute percent difference (MAPD) was 15.05%, with median APD 15.35% (IQR 4.65%–21.81%). Bland–Altman analysis showed a mean bias (Reader2–Reader1) of − 0.226 dL with limits of agreement − 1.917 to + 1.466 dL.

### Univariable analysis

At univariable testing (Table 2), the International Prognostic Index (IPI) emerged as the most robust predictor: each point increase raised the odds of 36-month progression by 66% (OR 1.66, *p* = 0.002) and increased the PFS hazard by 48% (HR 1.48, *p* < 0.001). Splenic involvement was similarly adverse: PET-positive spleen tripled the odds of 36-month progression (OR 3.49,* p* = 0.001) and doubled the hazard of relapse (HR 2.43, *p* = 0.001). Each decilitre increase in splenic volume raised the risk of progression by 9% (OR 1.09, *p* = 0.017) and the PFS hazard by 4% (HR 1.04, *p* = 0.006). Other adverse predictors included male sex (OR 2.32, *p* = 0.024; HR 1.99, *p* = 0.015) and presence of *B* symptoms (HR 1.75, *p* = 0.038). Age and HGBCL subtypes were not associated with adverse outcomes.

**Table 2:** Univariate logistic and Cox regression analyses for risk of progression/relapse and progression-free survival (PFS). Variables are listed with effect estimates, 95% confidence intervals, and two-sided *p* values

Variableᶜᵈ	Logistic OR (95% CI)ᵃ	p value	Cox HR (95% CI)ᵇ	*p* value
Sex (Male vs. Female)	2.32 (1.13–4.89)	0.023	1.99 (1.14–3.46)	0.015
Age at staging (years)	1.00 (0.98–1.03)	0.848	1.01 (0.98–1.03)	0.629
B symptoms (Yes vs. No)	1.94 (0.93–4.10)	0.080	1.75 (1.03–2.97)	0.038
IPI score (0–5)	1.66 (1.23–2.32)	0.001	1.48 (1.19–1.83)	< 0.001
Splenic PET positivity (Yes vs. No)	3.49 (1.64–7.66)	0.001	2.43 (1.43–4.12)	0.001
Baseline spleen volume (dL)	1.09 (1.02–1.19)	0.017	1.04 (1.01–1.08)	0.006
HGBCL (vs. DLBCL-NOS)	0.67 (0.14–2.69)	0.586	0.75 (0.23–2.43)	0.634
Other subtypes (vs. DLBCL-NOS)	4.02 (1.13–18.93)	0.045	2.77 (1.35–5.70)	0.006
Intensive therapy (vs. Reduced)	0.17 (0.01–1.41)	0.144	0.20 (0.06–0.64)	0.006
Standard therapy (vs. Reduced)	0.12 (0.01–0.74)	0.054	0.17 (0.07–0.41)	< 0.001

ᵃLogistic regression models assess the binary endpoint (progression/relapse within 36 months); effect size is odds ratio (OR)

ᵇCox models assess the time-to-event endpoint (PFS); effect size is hazard ratio (HR)

ᶜContinuous covariates (age, spleen volume, IPI) were entered as linear terms

ᵈCategorical covariates are shown versus the indicated reference category (Female; No B symptoms; DLBCL-NOS; Reduced therapy)

CI = confidence interval; OR = odds ratio; HR = hazard ratio; PFS = progression-free survival. Abbreviations introduced in (Table 1) (IPI, DLBCL-NOS, HGBCL, PFS, ECOG, LDH, IQR) apply here as well

### Multivariable analysis

**Fig. 2:**
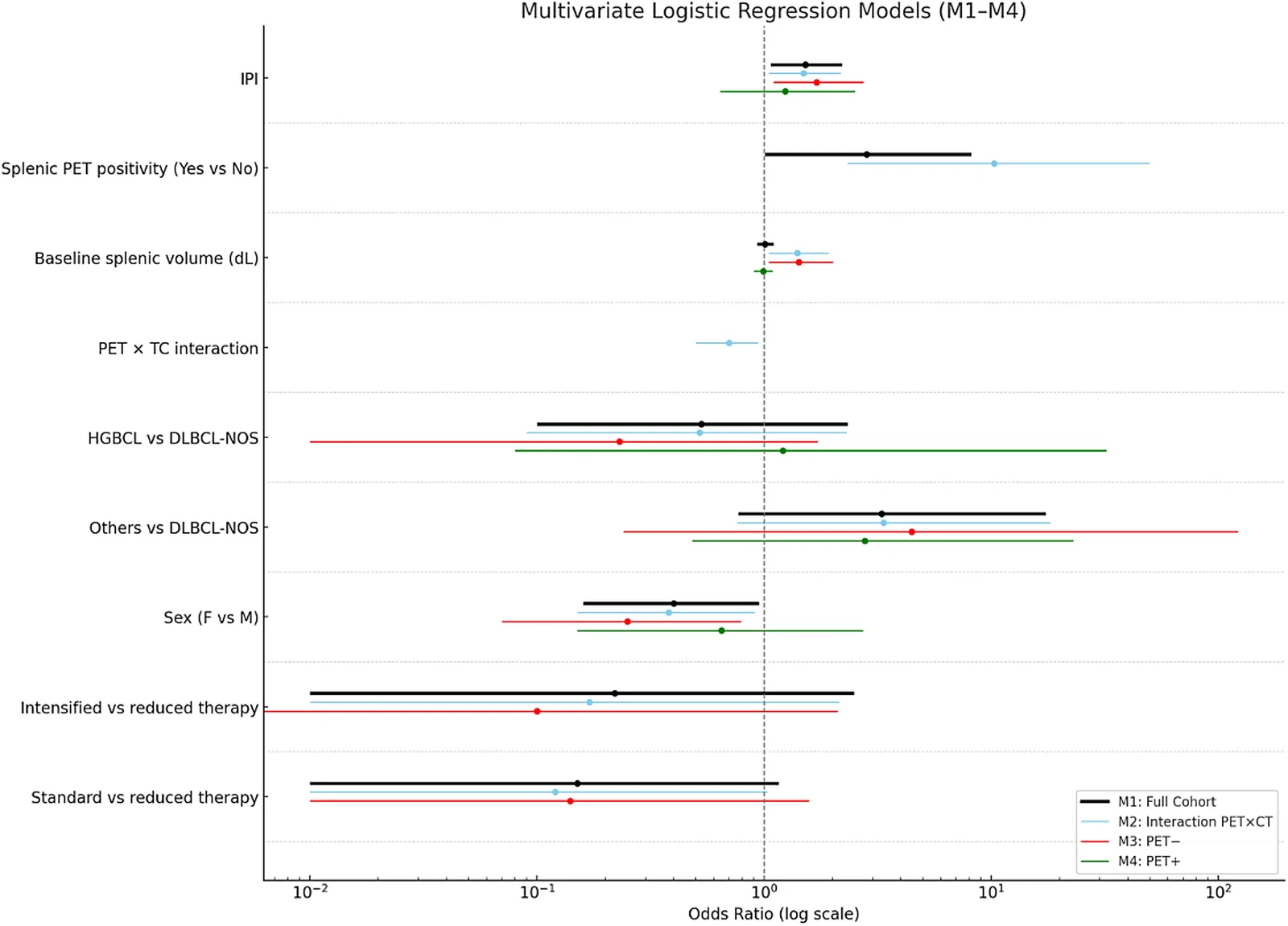
Adjusted odds ratios from multivariable logistic regression models. Forest plot of adjusted odds ratios (OR) with 95% confidence intervals (CI) for covariates included in multivariable logistic regression. Models: M1 = full cohort; M2 = full cohort + PET × CT interaction; M3 = PET-negative subgroup; M4 = PET-positive subgroup. OR = odds ratio; CI = confidence interval; PET = positron emission tomography; CT = computed tomography

**Fig. 3:**
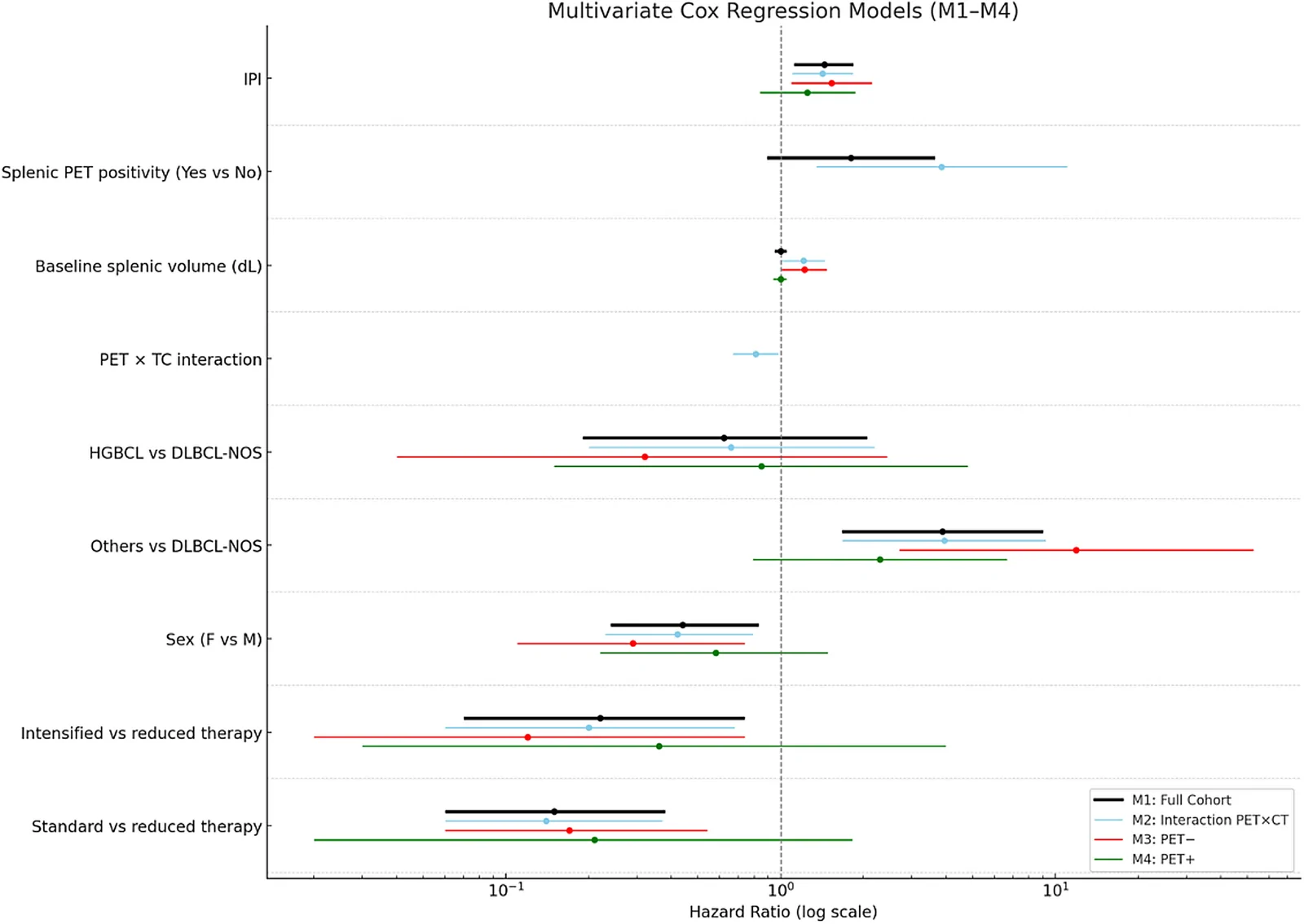
Adjusted hazard ratios from multivariable Cox models. Forest plot of adjusted hazard ratios (HR) with 95% confidence intervals (CI) for covariates included in multivariable Cox proportional hazards regression. Models: M1 = full cohort; M2 = full cohort + PET × CT interaction; M3 = PET-negative subgroup; M4 = PET-positive subgroup. HR = hazard ratio; CI = confidence interval

Four prespecified multivariable models were developed using both logistic and Cox frameworks (Tables 3 and 4, Figs. 2 and 3). In the full cohort (model M1), IPI remained an independent predictor of early progression (OR 1.52, *p* = 0.021) and PFS (HR 1.44,* p* = 0.004). PET-positive spleen was independently associated with the binary outcome (OR 2.83, *p* = 0.048), though it did not reach significance in the Cox model (HR 1.80, *p* = 0.103). Splenic volume showed no independent effect (*p* > 0.80), while female sex was protective (OR 0.40; HR 0.44). Treatment category, although forced into all models, had no effect on the binary outcome but was associated with better PFS for standard versus reduced-intensity therapy (HR 0.15).

**Table 3:** Multivariable logistic regression models (M1–M4) for risk of progression/relapse within 36 months. Models: M1 = full cohort; M2 = full cohort + PET × CT interaction; M3 = PET-negative only; M4 = PET-positive only. Dashes (—) denote covariates not evaluated in the respective model

Variableᵇ	M1 OR (95% CI)ᵃ	p value	M2 OR (95% CI)ᵃ	p value	M3 OR (95% CI)ᵃ	p value	M4 OR (95% CI)ᵃ	p value
IPI score	1.52 (1.07–2.20)	0.021	1.49 (1.05–2.18)	0.029	1.70 (1.10–2.74)	0.022	1.24 (0.64–2.51)	0.523
Splenic PET positivity (Yes vs. No)	2.83 (1.01–8.15)	0.048	10.27 (2.33–49.83)	0.002	–	–	–	–
Baseline Splenic Volume	1.01 (0.93–1.10)	0.880	1.40 (1.05–1.92)	0.024	1.42 (1.05–2.01)	0.027	0.99 (0.90–1.09)	0.848
PET × CT interactionᶜ	–	–	0.70 (0.50–0.94)	0.021	–	–	–	–
HGBCL versus DLBCL-NOS	0.53 (0.10–2.33)	0.415	0.52 (0.09–2.30)	0.404	0.23 (0.01–1.72)	0.212	1.21 (0.08–32.19)	0.892
Others versus DLBCL-NOS	3.29 (0.77–17.32)	0.123	3.36 (0.76–18.22)	0.125	4.45 (0.24–122.02)	0.300	2.77 (0.48–22.93)	0.283
Sex (F vs. M)	0.40 (0.16–0.95)	0.043	0.38 (0.15–0.91)	0.034	0.25 (0.07–0.79)	0.025	0.65 (0.15–2.72)	0.555
Intensified versus reduced therapy	0.22 (0.01–2.49)	0.262	0.17 (0.01–2.14)	0.209	0.10 (0.00–2.11)	0.169	3.5e–07 (NA)	0.995
Standard versus reduced therapy	0.15 (0.01–1.16)	0.110	0.12 (0.01–1.04)	0.089	0.14 (0.01–1.58)	0.147	1.2e–07 (NA)^d^	0.995

ᵃOdds ratios (OR) with 95% confidence intervals (CI) and two-sided Wald p values

ᵇReference categories: Male, DLBCL-NOS, Reduced therapy, No for PET positivity and B symptoms (where applicable)

ᶜPET × CT interaction = interaction between splenic PET positivity and baseline splenic volume (included only in M2)

^d^NA = Not applicable, CI not estimable due to sparse data

All abbreviations used in this table were defined previously (see Tables 1 and 2)

**Table 4:** Multivariable Cox regression models (M1–M4) for progression-free survival (PFS). Models: M1 = full cohort; M2 = full cohort + PET × CT interaction; M3 = PET-negative only; M4 = PET-positive only. Dashes (—) denote covariates not evaluated in the respective model

Variableᵇ	M1 HR (95% CI)ᵃ	p value	M2 HR (95% CI)ᵃ	p value	M3 HR (95% CI)ᵃ	p value	M4 HR (95% CI)ᵃ	p value
IPI score	1.44 (1.12–1.84)	0.004	1.42 (1.10–1.83)	0.007	1.53 (1.09–2.15)	0.014	1.25 (0.84–1.87)	0.264
Splenic PET positivity (Yes vs. No)	1.80 (0.89–3.64)	0.103	3.85 (1.35–11.02)	0.012	–	–	–	–
Baseline splenic volume	1.00 (0.95–1.05)	0.952	1.21 (1.01–1.45)	0.037	1.22 (1.01–1.47)	0.042	1.00 (0.94–1.05)	0.872
PET × CT interactionᶜ	–	–	0.81 (0.67–0.98)	0.033	–	–	–	–
HGBCL versus DLBCL-NOS	0.62 (0.19–2.06)	0.435	0.66 (0.20–2.20)	0.497	0.32 (0.04–2.44)	0.270	0.85 (0.15–4.78)	0.857
Others versus DLBCL-NOS	3.88 (1.67–9.01)	0.002	3.94 (1.68–9.23)	0.002	11.91 (2.70–52.55)	0.001	2.30 (0.79–6.67)	0.125
Sex (F vs. M)	0.44 (0.24–0.83)	0.010	0.42 (0.23–0.79)	0.006	0.29 (0.11–0.74)	0.009	0.58 (0.22–1.48)	0.252
Intensified versus reduced therapy	0.22 (0.07–0.74)	0.015	0.20 (0.06–0.68)	0.010	0.12 (0.02–0.74)	0.022	0.36 (0.03–3.99)	0.406
Standard versus reduced therapy	0.15 (0.06–0.38)	< 0.001	0.14 (0.06–0.37)	< 0.001	0.17 (0.06–0.54)	0.003	0.21 (0.02–1.82)	0.155

ᵃHazard ratios (HR) with 95% confidence intervals (CI) and two-sided Wald p values

ᵇReference categories: Male, DLBCL-NOS, Reduced therapy, No for PET positivity and B symptoms (where applicable)

ᶜPET × CT = interaction term between splenic PET positivity and baseline splenic volume (included only in M2)

All abbreviations used in this table have been defined previously (see Tables 1, 2 and 3)

Adding the PET × volume interaction (model M2) supported effect modification by metabolic status. In the primary Cox analysis (*N* = 124), the interaction term was statistically significant (HR 0.81, 95% CI 0.67–0.98; *p* = 0.033). When formally compared with the additive Cox model (M1), M2 provided a modest improvement in fit (likelihood-ratio *χ*^2^ = 4.04, df = 1; *p* = 0.045), with a small change in discrimination (ΔC =  + 0.004) and a modest AIC reduction (ΔAIC =  − 2.04), while BIC penalised the added complexity.

**Fig. 4:**
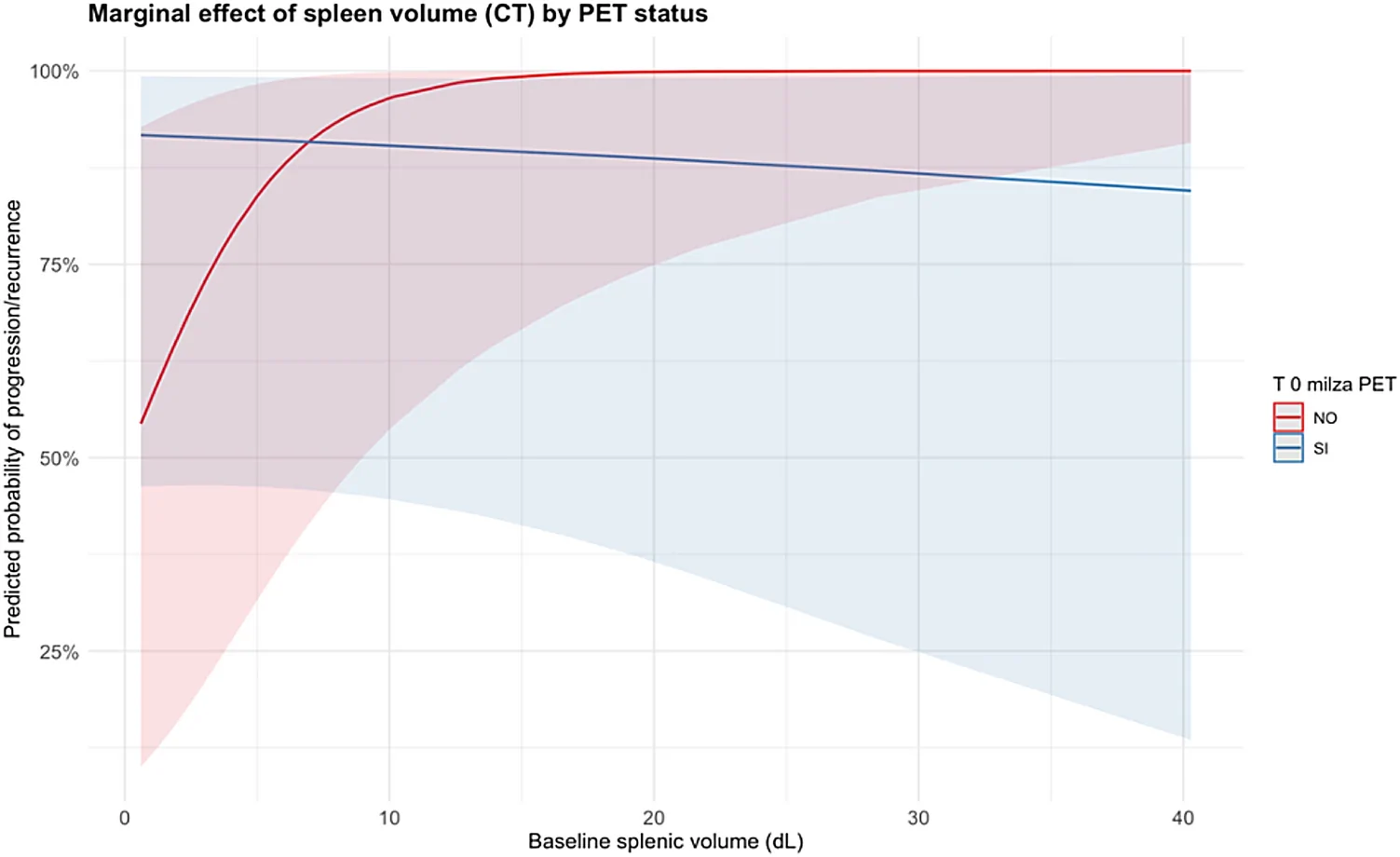
Marginal effect of splenic volume on relapse probability, stratified by PET status. Plot showing predicted probabilities of progression from Model M2, stratified by PET status. Curves show the effect of baseline splenic volume on relapse risk in PET-negative (red) and PET-positive (blue) patients; shaded areas indicate 95% confidence intervals. PET = positron emission tomography

Splenic volume was relevant only in the absence of metabolic activity. In PET-negative cases, relapse risk increased with volume (Fig. 4), while in PET-positive cases, volume had no additive effect (net OR ≈ 0.98; HR ≈ 0.98). All logistic models were well calibrated (Hosmer–Lemeshow *p* > 0.05) and had low multicollinearity (VIF < 3.5). Discrimination was good (AUC-ROC 0.78–0.82; AUC-PR 0.70–0.78). Cox models met proportional hazards assumptions (global Schoenfeld *p* > 0.30). Concordance was high in M1–M3 (C 0.737–0.772) and modest in M4 (C 0.637). Full diagnostics and ROC/PR curves are shown in (Fig. S4), (Table S1 and S2).

In the PFS sensitivity analysis including event-free patients censored before 36 months (complete-case *N* = 145), effect directions were preserved but the interaction attenuated to borderline (HR 0.83, 95% CI 0.69–1.01; *p* = 0.057), and the incremental fit gain was smaller (likelihood-ratio *p* = 0.070; ΔC =  + 0.005; ΔAIC =  − 1.29). In PET-stratified Cox models, spleen volume remained directionally adverse in PET-negative patients but was borderline (HR 1.21, 95% CI 1.00–1.47; *p* = 0.053), whereas volume was null in PET-positive patients (HR 1.00, 95% CI 0.94–1.05; *p* = 0.878). Full sensitivity results are reported in Table S4 and model-comparison metrics in (Table S5).

### Splenic volume thresholds

Exploratory analyses identified two data-driven spleen volume cut-offs (Fig. S5). ROC analysis for 36-month progression indicated 4.11 dL (AUC 0.68; sensitivity 52%; specificity 78%). For PFS, the maximally selected rank statistic yielded 5.29 dL (log-rank *M* = 3.56; Monte Carlo *p* = 0.006). These cut-offs were not used in final models and should be interpreted as hypothesis-generating.

## Discussion

Our study evaluated the prognostic value of baseline splenic involvement in newly diagnosed DLBCL, assessed by metabolic imaging (PET) and anatomical volume (CT). Clinical and imaging variables were analysed both individually and with multivariable models to assess their combined and interactive effects. The International Prognostic Index (IPI) remained the most robust predictor in both univariable and multivariable analyses, consistent with prior studies [7, 23]. Therefore, imaging biomarkers may refine risk stratification but do not replace established clinical indices. In the PET-positive subgroup, IPI did not retain statistical significance for either endpoint, which is more plausibly explained by limited power and heterogeneity (*n* = 45) and non-significant global models than by a true lack of prognostic relevance; although partial overlap between PET findings and IPI components also cannot be excluded. Male sex was associated with increased relapse risk and shorter PFS, in line with reports of poorer outcomes in men in large population cohorts, potentially reflecting hormonal, immune, or pharmacokinetic mechanisms [24]. Baseline splenic PET positivity independently predicted early progression in logistic regression (OR 2.83), although this association did not reach statistical significance in time-to-event modelling.

**Fig. 5:**
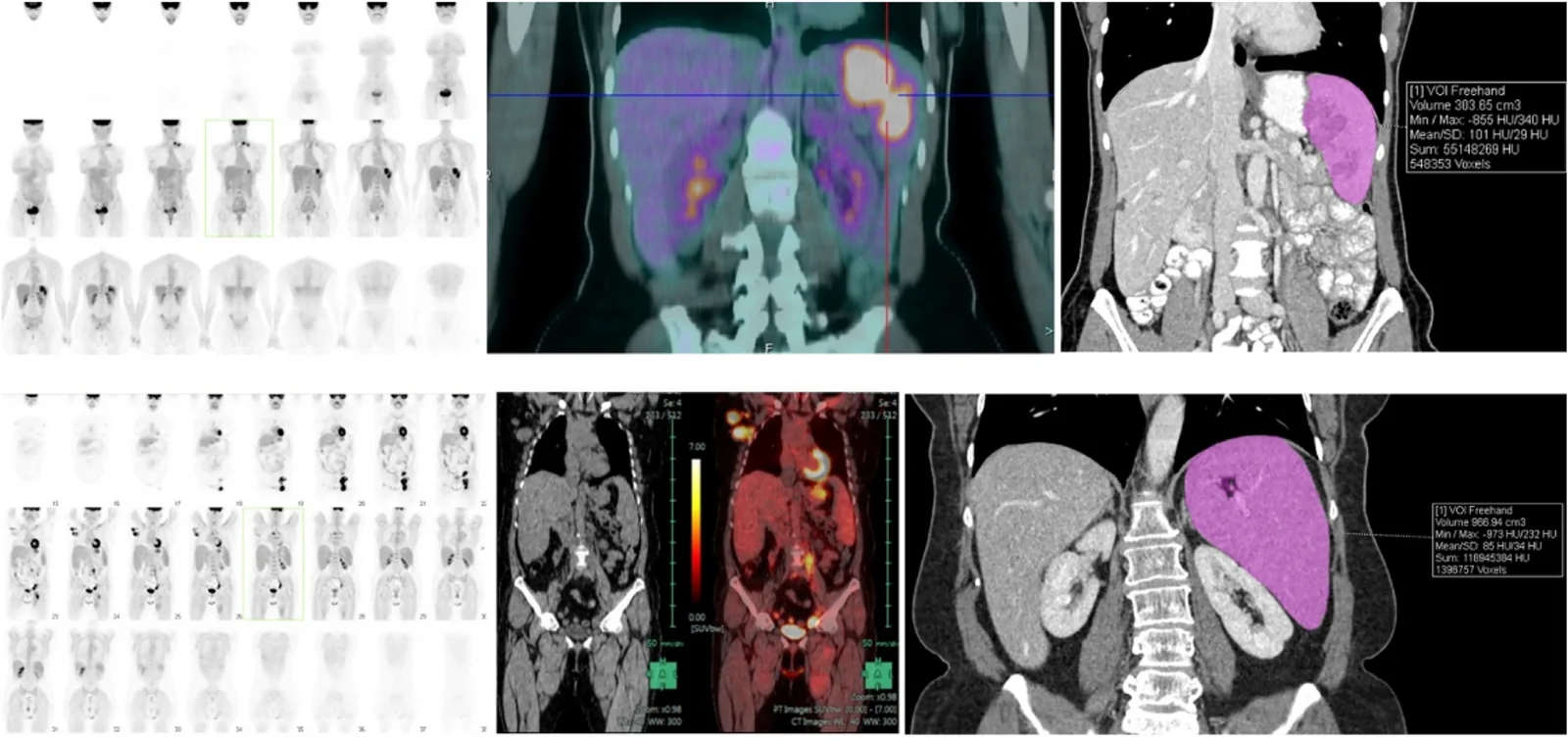
Baseline PET/CT and contrast-enhanced CT (CECT) in two patients with DLBCL. (**a**–**c**) PET-positive spleen without splenomegaly: (**a**) whole-body PET maximum intensity projection, (**b**) coronal PET/CT fused image, (**c**) coronal CECT with spleen segmentation showing normal volume (3.03 dL). (**d**–**f**) PET-negative spleen with marked splenomegaly: (**d**) whole-body PET maximum intensity projection, (**e**) coronal PET/CT fused image, (**f**) coronal CECT with spleen segmentation showing splenic enlargement (9.67 dL). PET = positron emission tomography; CT = computed tomography; CECT = contrast-enhanced CT; DLBCL = diffuse large B cell lymphoma

CT-derived splenic volume alone was not prognostic. However, the PET × volume interaction suggests effect modification by splenic metabolic status: splenomegaly appeared prognostically relevant mainly among PET-negative patients, whereas it carried little incremental signal in PET-positive cases. Importantly, the added value of the interaction in Cox models was modest and attenuated in sensitivity analyses; therefore, this pattern should be regarded as hypothesis-generating. Representative cases with discordant PET and volume profiles illustrate this complementary risk pattern (Fig. 5).

This conditional interpretation is biologically coherent. When splenic FDG uptake is present, it likely reflects metabolically active disease and/or disseminated tumour burden and may dominate risk assessment, consistent with prior evidence [7, 8]. Conversely, in PET-negative patients, splenomegaly may capture adverse biology not detected by a liver-referenced visual criterion—potentially reflecting low-grade diffuse infiltration, reactive hyperplasia, or a permissive microenvironment. While metabolic splenic involvement without splenomegaly has been described in lymphoma imaging [25], the context-dependent prognostic role of CT-derived splenic volume and its interaction with PET is novel for risk stratification. Wang et al. (2023) [7] reported differences in splenic size and focal lesions by recurrence status, but only focal lesions were independently prognostic in multivariable analyses. Our results help reconcile these inconsistencies by suggesting that volume-based splenic enlargement may carry prognostic information primarily when metabolic involvement is not evident.

Placed in the broader landscape of PET-based prognostic modelling in DLBCL, this finding aligns with the shift towards systemic imaging risk markers. Baseline whole-body PET-derived tumour burden measures—particularly total metabolic tumour volume (TMTV/MTV) and total lesion glycolysis (TLG)—have been repeatedly associated with outcome and are increasingly used to refine PET-informed risk stratification beyond single-site parameters [26, 27] PET-driven composite indices integrating PET burden with clinical variables and PET radiomics signatures capturing heterogeneity have also been proposed to improve discrimination over clinical scores alone in selected cohorts [28, 29]. Within this framework, splenic metrics may be interpreted as part of a broader systemic imaging signature rather than an isolated organ-based parameter.

Several non-mutually-exclusive mechanisms may underlie the association between splenomegaly and adverse outcome in PET-negative patients. First, a visual spleen-to-liver comparison may be insensitive to diffuse, low-contrast infiltration [25]. Second, FDG uptake is associated with proliferative activity—baseline SUVmax correlates with Ki-67 in DLBCL [30], so low-proliferation infiltrates may evade PET detection while still contributing to splenic enlargement. Third, tumour-driven cytokine signalling may promote extramedullary haematopoiesis (EMH) with expansion of myeloid progenitors and immunosuppressive myeloid-derived suppressor cells (MDSCs), potentially fostering immune escape [31, 32]. Elevated MDSCs in newly diagnosed DLBCL have been linked to higher tumour burden and inferior survival [33], supporting the hypothesis that EMH-associated splenomegaly could mark an adverse systemic environment in PET-negative disease.

The exploratory thresholds identified (4.11 dL for relapse prediction and 5.29 dL for PFS) closely match published volumetric definitions of massive splenomegaly (e.g. 4.31 dL in Linguraru et al.) [34]. This concordance supports biological plausibility and reinforces the value of volumetric over linear assessment for clinically meaningful stratification, as a commonly used linear cut-off (13 cm) showed limited diagnostic performance in Berzaczy et al. (2019) [35].

## Limitations

These findings should be interpreted in the light of several limitations. The retrospective single-centre design may introduce selection bias and limits generalisability, warranting external validation. Excluding event-free patients censored before 36 months was necessary to classify the fixed 36-month endpoint but may introduce survivor bias; we therefore repeated PFS Cox analyses including these 28 patients as right-censored at last contact, with directionally consistent estimates and attenuation of the PET × volume term to borderline. The number of events per variable was borderline (56 events overall; PET-positive subgroup *n* = 45), limiting precision and making interaction terms particularly sensitive to overfitting; accordingly, the PET × volume interaction should be considered exploratory and requires independent validation in accordance with TRIPOD principles for prognostic modelling [36].

Splenic volume was segmented semi-automatically by two readers with adjudication for > 5% discrepancy; reproducibility assessed on pre-adjudication measurements was good (ICC(2,1) = 0.912), but residual variability (MAPD≈15%) may have attenuated volume–outcome associations. Splenic PET positivity was derived from a single baseline clinical staging report, precluding inter-reader agreement assessment for the binary classification. Finally, semi-quantitative spleen-to-liver SUV ratios were not analysed because reliable quantitative comparability in a multi-scanner retrospective cohort over a long study window would require harmonised acquisition and reconstruction settings, cross-calibration, and consistent uptake time in accordance with EANM/EARL frameworks [37, 38].

The data-driven thresholds (4.11 dL and 5.29 dL) were hypothesis-generating and not incorporated in multivariable models. Finally, the binary endpoint was limited to 36 months—so late events were only captured in the PFS analysis.

## Conclusions

In conclusion, splenic PET positivity remains a dominant marker of disease burden and aggressiveness, whereas CT-based splenic volume adds conditional prognostic value in PET-negative patients. This work identifies a distinct high-risk phenotype—PET-negative DLBCL with pronounced splenomegaly—where CT volumetry captures adverse biology not detected by metabolic imaging and may warrant intensified follow-up. PET-negative patients with marked splenomegaly may represent a subgroup warranting closer surveillance and earlier clinical reassessment than typically planned for PET-negative staging, including a lower threshold for repeat imaging in the presence of symptoms or laboratory abnormalities. Future validation of this conditional dual-signal model, together with integration of advanced PET metrics and radiomic profiling [30, 39–42], could enhance risk-adapted care and improve clinical trial stratification.

## Supplementary Information

Below is the link to the electronic supplementary material.Supplementary file1 (DOCX 940 KB) 

## Abbreviations

DLBCL: Diffuse large B cell lymphoma
PET: Positron emission tomography
FDG: Fluorodeoxyglucose
CT: Computed tomography
IPI: International Prognostic Index
PFS: Progression-free survival
OR: Odds ratio
HR: Hazard ratio
CI: Confidence interval
