# Supplementary material for: Splenic FDG PET uptake and CT volume as prognostic biomarkers in diffuse large B cell lymphoma
Source: Radiol Med. 2026 Jun 20;131(8):1292–304. doi: 10.1007/s11547-026-02233-w (PMC13526055; doi:10.1007/s11547-026-02233-w)
Supplement: Supplementary file 1 [file 11547_2026_2233_MOESM1_ESM.docx]

**Electronic Supplementary Material**

**Figure S1. Distribution of baseline clinical, laboratory, and imaging variables by relapse status**

Each panel displays the distribution of baseline variables stratified by relapse within 36 months. Categorical variables are shown as bar plots with percentages by relapse status. Ordinal and continuous variables are shown as boxplots with overlaid data points. Titles report the statistical test and corresponding p value.

**Figure S2. Kaplan–Meier estimate of progression-free survival (PFS).**

The curve represents the probability of remaining free from disease progression or relapse over time since baseline staging. Shaded areas represent 95% confidence intervals.

**Figure S3. Baseline splenic volume stratified by PET positivity**

Comparison of splenic volume measured on baseline CT imaging in patients with PET-positive vs PET-negative spleen. PET positivity was associated with substantially increased splenic volume (U=612·5; p<0·0001).

**Figure S4. Discriminative performance of logistic models**

Left panel: Receiver operating characteristic (ROC) curves for each model. Right panel: Precision–recall (PR) curves. Colour coding denotes model identity: red = M1 (full cohort), blue = M2 (interaction PET × CT), green = M3 (PET−), purple = M4 (PET+). Area under the curve (AUC) and AUCPR values are shown in the legend.

**Figure S5. Threshold analysis of baseline splenic volume**

(a) ROC curve for the binary outcome of progression or relapse within 36 months, identifying an optimal baseline splenic volume threshold of 4.11 dL (AUC = 0.68). (b) Kaplan–Meier estimates of PFS stratified by splenic volume using the 5.29 dL cut-off, showing significantly reduced survival in patients above the threshold.

**Table S1. Metrics for multivariable logistic regression models**

| **Logistic Regression Model Metrics (M1–M4)** | | | | |
| --- | --- | --- | --- | --- |
| **Metric** | **M1** | **M2** | **M3** | **M4** |
| N (observations) | 124 | 124 | 79 | 45 |
| Residual deviance | 139.45 | 133.75 | 74.68 | 55.36 |
| AIC | 157.45 | 153.75 | 90.68 | 71.36 |
| McFadden R² | 0.18 | 0.22 | 0.26 | 0.13 |
| Hosmer-Lemeshow p | >0.05 | >0.05 | >0.05 | >0.05 |
| VIF max | ≈ 2.9 | ≈ 3.2 | ≈ 2.4 | ≈ 2.6 |
| AUC ROC (95% CI) | 0.80 (0.72–0.88) | 0.82 (0.74–0.89) | 0.78 (0.70–0.86) | 0.78 (0.69–0.86) |
| AUC PR (95% CI) | 0.77 (0.66–0.91) | 0.78 (0.68–0.90) | 0.71 (0.62–0.80) | 0.70 (0.61–0.78) |
| Accuracy (95% CI) | 0.72 (0.63–0.88) | 0.75 (0.66–0.89) | 0.77 (0.70–0.86) | 0.75 (0.69–0.86) |
| Sensitivity (95% CI) | 0.69 (0.60–0.91) | 0.71 (0.62–0.91) | 0.73 (0.63–0.92) | 0.73 (0.62–0.91) |
| Specificity (95% CI) | 0.72 (0.60–0.91) | 0.73 (0.62–0.91) | 0.69 (0.60–0.81) | 0.68 (0.59–0.82) |
| PPV (95% CI) | 0.71 (0.60–0.91) | 0.74 (0.63–0.91) | 0.74 (0.63–0.92) | 0.75 (0.64–0.92) |
| NPV (95% CI) | 0.70 (0.60–0.83) | 0.71 (0.61–0.84) | 0.68 (0.62–0.81) | 0.67 (0.61–0.82) |
| F1 score (95% CI) | 0.66 (0.51–0.80) | 0.67 (0.56–0.82) | 0.66 (0.49–0.78) | 0.64 (0.48–0.78) |

Model performance was assessed using residual deviance, Akaike Information Criterion (AIC), McFadden’s pseudo R², and the Hosmer–Lemeshow goodness-of-fit test. Multicollinearity was evaluated via the maximum variance inflation factor (VIF). Discrimination and classification performance were quantified using the area under the ROC curve (AUC ROC), precision–recall curve (AUC PR), accuracy, sensitivity, specificity, positive predictive value (PPV), negative predictive value (NPV), and F1 score, all reported with 95% confidence intervals (CI). Estimates were computed via 1000 bootstrap replicates.

**Table S2. Metrics for multivariable Cox proportional hazards models**

| **Cox Proportional Hazards Model Metrics (M1–M4)** | | | | |
| --- | --- | --- | --- | --- |
| **Metric** | **M1** | **M2** | **M3** | **M4** |
| N (observations) | 124 | 124 | 79 | 45 |
| Concordance index (C) | 0.737 | 0.742 | 0.772 | 0.637 |
| Likelihood Ratio test p-value | 4.8e-07 | 2.1e-07 | 4.1e-06 | 0.558 |
| Wald test p-value | 3.7e-07 | 4.9e-07 | 1.5e-05 | 0.501 |
| Score test p-value | 6.3e-09 | 5.8e-09 | 2.0e-09 | 0.396 |
| Schoenfeld test (global p) | 0.45 | 0.48 | 0.34 | 0.30 |

Discriminative ability was evaluated using the concordance index (C). Global model significance was tested using the likelihood ratio, Wald, and score (log-rank) tests. The proportional hazards assumption was assessed using the global Schoenfeld residual test.

**Table S3. Classification of First-Line Treatment Regimens by Intensity**

| **Treatment Category** | **Abbreviation** | **Full Regimen Name** |
| --- | --- | --- |
| **Standard-intensity** | R-CHOP | Rituximab, Cyclophosphamide, Doxorubicin, Vincristine, Prednisone |
|  | R-COMP | Rituximab, Cyclophosphamide, Liposomal Doxorubicin, Vincristine, Prednisone |
| **Intensified** | GMALL | German Multicenter Protocol for Adult Acute Lymphoblastic Leukemia (adapted) |
|  | HyperCVAD | Hyperfractionated Cyclophosphamide, Vincristine, Doxorubicin, Dexamethasone |
|  | R-CODOX/M-IVAC | Rituximab, Cyclophosphamide, Vincristine, Doxorubicin, Methotrexate / Ifosfamide, Etoposide, Cytarabine |
|  | R-DHAOx | Rituximab, Dexamethasone, Cytarabine, Oxaliplatin |
| **Reduced-intensity** | R-mini-CHOP | Reduced-dose Rituximab, Cyclophosphamide, Doxorubicin, Vincristine, Prednisone |
|  | R-mini-COMP | Reduced-dose Rituximab, Cyclophosphamide, Liposomal Doxorubicin, Vincristine, Prednisone |
|  | R-CVP | Rituximab, Cyclophosphamide, Vincristine, Prednisone |

First-line treatment regimens were categorised into three groups based on therapeutic intensity: standard, intensified, and reduced-intensity. The table lists each regimen within its respective category alongside the corresponding full protocol name.

**Table S4. Sensitivity multivariable Cox models (M1–M4) for progression-free survival (PFS)**

| **Variable** | **M1** | **M2** | **M3** | **M4** |
| --- | --- | --- | --- | --- |
| IPI (per +1) | 1.46 (1.15–1.86); p=0.002 | 1.42 (1.11–1.81); p=0.005 | 1.56 (1.12–2.17); p=0.008 | 1.24 (0.83–1.85); p=0.294 |
| Spleen PET positivity (Yes vs No) | 1.90 (0.94–3.83); p=0.075 | 3.80 (1.32–10.92); p=0.013 | — | — |
| Baseline splenic volume (dL, per +1) | 1.00 (0.95–1.05); p=0.958 | 1.18 (0.99–1.42); p=0.064 | 1.21 (1.00–1.47); p=0.053 | 1.00 (0.94–1.05); p=0.878 |
| PET×Volume interaction (Yes×dL) | — | 0.83 (0.69–1.01); p=0.057 | — | — |
| HGBCL vs DLBCL-NOS | 0.61 (0.18–2.06); p=0.427 | 0.68 (0.20–2.31); p=0.540 | 0.31 (0.04–2.38); p=0.259 | 0.93 (0.17–5.24); p=0.939 |
| Others vs DLBCL-NOS | 4.32 (1.86–10.02); p=<0.001 | 4.26 (1.83–9.96); p=<0.001 | 12.66 (2.84–56.48); p=<0.001 | 2.52 (0.87–7.31); p=0.089 |
| Sex (Female vs Male) | 0.39 (0.21–0.73); p=0.003 | 0.37 (0.20–0.69); p=0.002 | 0.25 (0.10–0.64); p=0.004 | 0.50 (0.20–1.29); p=0.152 |
| Intensified vs reduced therapy | 0.20 (0.06–0.66); p=0.008 | 0.16 (0.05–0.55); p=0.004 | 0.09 (0.02–0.56); p=0.009 | 0.32 (0.03–3.54); p=0.352 |
| Standard vs reduced therapy | 0.15 (0.06–0.37); p=<0.001 | 0.14 (0.05–0.35); p=<0.001 | 0.16 (0.05–0.51); p=0.002 | 0.20 (0.02–1.78); p=0.149 |

Prespecified multivariable Cox proportional-hazards models for PFS are reported, re-estimated in a sensitivity cohort that additionally includes event-free patients with follow-up shorter than 36 months. Models are defined as: M1 (full cohort, additive main effects), M2 (full cohort plus PET×CT interaction), M3 (PET-negative subgroup), and M4 (PET-positive subgroup). Effect estimates are hazard ratios (HR) with 95% confidence intervals (CI) and two-sided Wald p values. Reference categories are: Male, DLBCL-NOS, and Reduced-intensity therapy; for PET status (where applicable), the reference is PET-negative. The PET×CT term denotes the interaction between splenic PET positivity and baseline CT-derived splenic volume (included only in M2).

**Table S5. Incremental value of the PET×CT interaction term for PFS: comparison of the additive (M1) and interaction (M2) Cox models in the primary analysis and sensitivity cohorts**

| **Cohort** | **N** | **Events** | **LRT χ² (df=1)** | **LRT p** | **ΔAIC (M2–M1)** | **ΔBIC (M2–M1)** | **C-index M1** | **C-index M2** | **ΔC-index (M2–M1)** |
| --- | --- | --- | --- | --- | --- | --- | --- | --- | --- |
| Primary analysis cohort | 124 | 56 | 4.036 | 0.045 | -2.04 | 0.78 | 0.737 | 0.742 | 0.004 |
| Sensitivity cohort | 145 | 56 | 3.285 | 0.070 | -1.29 | 1.69 | 0.755 | 0.760 | 0.005 |

We assessed whether adding the PET×CT interaction term provides incremental prognostic information beyond the corresponding additive model (main effects only). For each cohort, we report the sample size and number of PFS events, the likelihood-ratio test (LRT) for nested models (M2 vs M1; 1 additional parameter), information criteria (AIC and BIC), and Harrell’s concordance index (C-index). ΔAIC, ΔBIC, and ΔC-index indicate the change from M1 to M2, with negative ΔAIC supporting improved penalised fit and positive ΔBIC indicating limited support under the stronger complexity penalty. C-index changes quantify any incremental gain in discrimination.
